# Morgagni hernia presented as sudden dyspnea in 70 years old man: a case report

**DOI:** 10.11604/pamj.2022.41.42.32763

**Published:** 2022-01-17

**Authors:** Menawar Dajenah, Anessa Thabet, Faisal Ahmed

**Affiliations:** 1 Department of General Surgery, School of Medicine, Ibb University of Medical Science, Ibb, Yemen,; 2 Department of Gynecology, School of Medicine, Ibb University of Medical Science, Ibb, Yemen,; 3 Urology Research Center, Al-Thora General Hospital, Department of Urology, School of Medicine, Ibb University of Medical Science, Ibb, Yemen

**Keywords:** Adult, Morgagni diaphragmatic hernia, respiratory failure, surgery, case report

## Abstract

Diaphragmatic hernia is a structural defect caused by inadequate fusion of the pleuroperitoneal membrane of the diaphragm, allowing peritoneal viscera to protrude into the pleural cavity. The occurrence of Morgagni hernia in the adult is infrequent and almost asymptomatic. Symptomatic cases are even rarer, with a wide range of respiratory and gastrointestinal manifestations that make it difficult to diagnose. We present the case of a 70-year-old man with unexpected onset abdominal pain and respiratory distress. The chest computed tomography scan showed the right-side diaphragmatic Morgagni hernia. The defect was corrected through open surgical repair without complications. Within five months of the procedure, a follow-up radiograph revealed full recovery. This case should alert physicians to consider this diagnosis when faced with an unexpected manifestation of Morgagni hernia.

## Introduction

Diaphragmatic hernia is a hereditary congenital disorder characterized by abnormal diaphragmatic growth, which affects one out of 2500 neonates and has an overall survival of 67%. This condition is caused by a diaphragmatic tunnel that lets peritoneal viscera protrude into the pleural cavity [1]. Morgagni diaphragmatic hernia occurrence in adults is exceedingly rare. Mostly it is asymptomatic in adults, and it is frequently discovered as an incidental finding on chest X-ray or Computerized Tomography (CT) scan [2]. Symptomatic Morgagni hernia in an adult is even rarer, with a wide range of gastrointestinal and respiratory symptoms, making the diagnosis even more challenging [3, 4]. We present a case of right-sided Morgagni hernia in a 70-year-old male who presents with sudden dyspnea and was successfully treated with open surgery.

## Patient and observation

**Patient information:** a 70-year-old man with a complaint of acute onset abdominal pain and respiratory difficulties. His abdominal pain was localized in the epigastric area, puncturing type, not radiated, and worsened with lying down. He had a history of repeated postprandial vomiting and hiccups, which were progressive and not improved by medications for one year. The patient had no history of respiratory distress, asthma, gastrointestinal disease, or trauma to the abdomen. The patient is a case of chronic hypertension on medications.

**Clinical findings:** regarding the physical examination, the pulse rate was 72 beats per minute, the respiratory rate: 30 per min, the O_2_ saturation: 90% with a nasal cannula, and the oral temperature: 37.8°C. There was less air entrance in the right lung and the heart sounds were weekly noticed on the left lung. The abdomen was scaphoid without any palpated mass or tenderness. Additionally, the bowel sound was detected in the right thoracic cavity.

**Figure 1:**
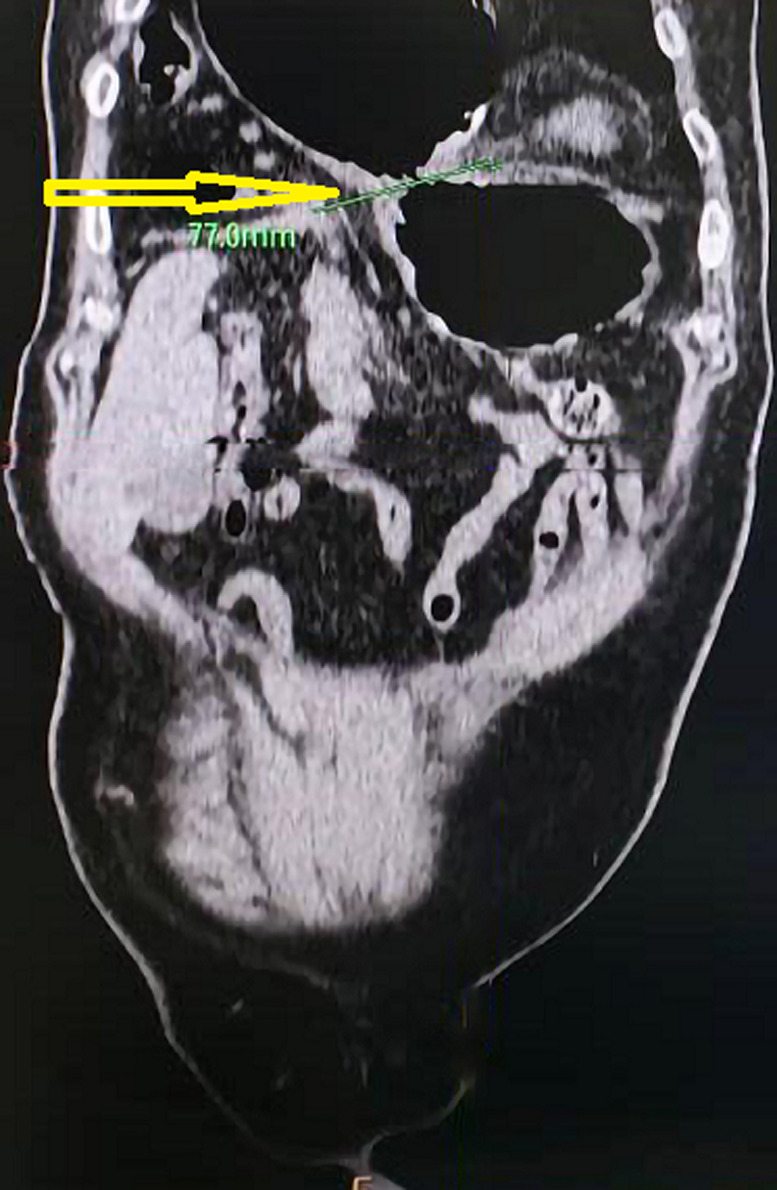
computed tomography scan showed the Morgagni hernia (arrow)

**Diagnostic assessment:** blood tests revealed a total white blood cell count of 15 ×10^3^/ml with moderate leukocytosis and hemoglobin: 12.4 g/dl; all other blood investigations were within the normal range. Metabolic acidosis was discovered by an arterial blood gas test, with serum lactate levels of 3.3 mmol/L. According to the chest X-ray, the stomach was seen in the right chest cavity. Chest and abdomen Computed Tomography (CT) scans were carried out for validating the diagnosis of right diaphragmatic hernia of the omentum, bowel loop, stomach, and transverse colon about 8 cm and collapse of the underlying lung and shift of the mediastinum to the left side (Figure 1).

**Figure 2:**
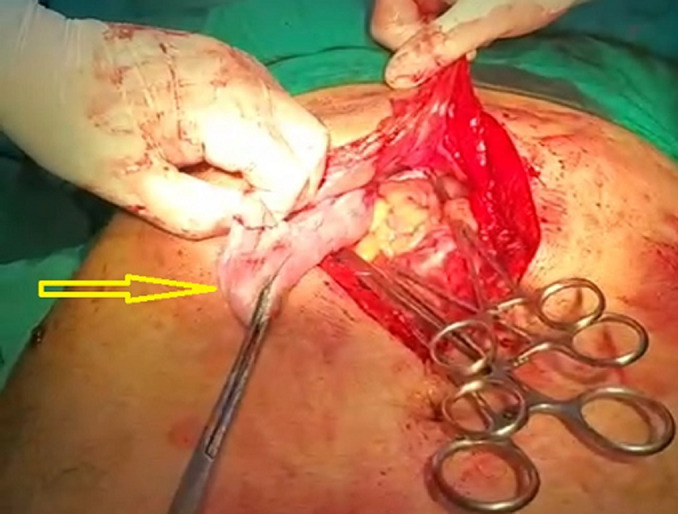
intraoperative viewing of the Morgagni herniation with peritoneal content (arrow)

**Figure 3:**
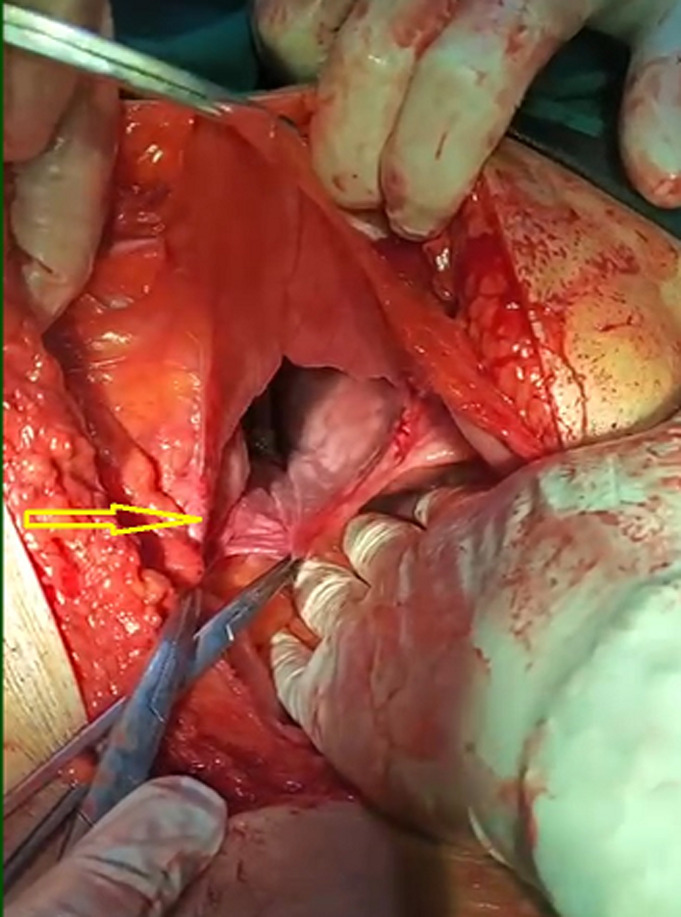
intraoperative view of Morgagni herniation after decrement of the bowel components to the peritoneal cavity (arrow)

**Therapeutic interventions:** via general anaesthesia with supine position, the abdomen was opened through the right subcostal incision, where a right diaphragmatic defect of around 8 cm in diameter (Figure 2). The existence of the small bowel, transverse colon, and stomach within the chest cavity was observed and necessitated a decrement of the components to the peritoneal cavity after confirming viability by inspection (Figure 3). In addition, the hernial defect was repaired with a non-absorbable suture. After confirmation of diaphragmatic consistency, the chest tube was placed and ended without intraoperative complications.

**Follow-up and outcome:** a control chest X-ray was performed 24 hours after the operation, which revealed a cardiac silhouette in a proper position and good expansion of the right lung and left lung infiltration. The patient was discharged from the hospital on the 11^th^ postoperative day without complications. After a 5-month follow-up, the patient was in good condition, and the chest X-ray was normal.

**Patient perspective:** the patient was happy with the successful outcome of the surgery.

**Informed consent:** a written informed consent was obtained from the patient for participation in our study.

## Discussion

Hunter and Mc Cauley published the first explanation of the pathophysiology of congenital diaphragmatic defects (CHD) in 1754, defining it as the protrusion of peritoneal organs into the pleural cavity due to genetic or environmental triggers. The Morgagni hernia defect is the fewest type of CHD, with a frequency between 1% to 5.1% [5, 6].

Morgagni hernia is more frequently occurs on the right side, which happens because widespread pericardial attachments on the left provide additional support. It is seen in women more than men and is associated with extra congenital anomalies [6]. The hernia's components are mainly the omentum, liver, colon, or small bowel, either alone or in a mixture with one of the other components. Many patients are symptomless, while vague epigastric frustration is the only clinical manifestation in many cases. Even though it is a congenital disorder, many cases do not manifest until later. This is thought to be due to the existence of a sac that limits herniation. A preceding event, such as abdominal trauma or increased intra-peritoneal pressure, causes the sac to rupture, resulting in herniation and the presence of symptoms. Another theory is that herniation occurs early, but symptoms appear once the gut becomes compromised [5].

The clinical features of Morgagni hernia comprise a wide range, in which gastrointestinal symptoms (such as vomiting and abdominal pain) and lung problems (such as shortness of breath, cough, and tachypnea) could appear alone or together. An excavated abdomen (hallow-out area), displacement of heart sounds, and auscultation of bowel movements in the chest may be seen [7].

Morgagni hernia is frequently detected incidentally on a plain radiograph as homogeneous masses in the right cardio phrenic angle. However, that chest radiography may be normal, particularly in infrequent herniation. When the diagnosis is ambiguous, the spiral chest CT scan, plain radiography after placement of a nasogastric tube, or Barium swallow could be performed to rule out the occurrence of Morgagni hernia [8]. The existence of a pleural mass with fat density in a CT scan representing herniated omentum or a combination of omentum and a peritoneal organ confirms the diagnosis [7]. According to a senior radiologist's review of his CT scan, our patient's diagnosis was carried out.

Once established, the environment determines treatment. Unless there is a sign of bowel compromise, surgical intervention can be postponed pending appropriate radiographic images that are obtainable [8]. Surgical intervention can be approached in a variety of ways. There have been reports of both transthoracic and transabdominal techniques. There have also been numerous reports of surgical laparoscopy repairs of Morgagni's hernias in the literature. Primary closure is generally used for more minor defects, but mesh may be required for more significant defects [7]. The postoperative complications include pericardial tamponade, lung embolus, collapsed lung, wound seroma, and haematoma [9]. Our patient was passed the postoperative period without any complications.

## Conclusion

The absence of usual clinical presentations in cases of Morgagni´s hernias affects the diagnosis of this defect. A misdiagnosis can result in bleeding, bowel obstruction, or strangulation, which needs early surgical intervention. Morgagni´s hernias should be considered through different methods of diagnosis and radiologic imaging studies. Additionally, open surgical repair was the best option for our patients.

## References

[1] Klein J, Sirota M (2017). Congenital Diaphragmatic Hernia. N Engl J Med.

[2] Herling A, Makhdom F, Al-Shehri A, Mulder DS (2014). Bochdalek hernia in a symptomatic adult. Ann Thorac Surg.

[3] Portelli M, Bugeja M, Cini C (2021). Left-Sided Bochdalek´s Hernia in a Young Adult: A Case Report and Literature Review. Surg J (N Y).

[4] Akita M, Yamasaki N, Miyake T, Mimura K, Maeda E, Nishimura T (2020). Bochdalek hernia in an adult: two case reports and a review of perioperative cardiopulmonary complications. Surg Case Rep.

[5] Patial T, Negi S, Thakur V (2017). Hernia of Morgagni in the Elderly: A Case Report. Cureus.

[6] Nasr A, Fecteau A (2009). Foramen of Morgagni hernia: presentation and treatment. Thorac Surg Clin.

[7] Arikan S, Dogan MB, Kocakusak A, Ersoz F, Sari S, Duzkoylu Y (2018). Morgagni's Hernia: Analysis of 21 Patients with Our Clinical Experience in Diagnosis and Treatment. Indian J Surg.

[8] Modi M, Dey AK, Mate A, Rege S (2016). Strangulated Morgagni's Hernia: A Rare Diagnosis and Management. Case Rep Surg.

[9] Katsaros I, Katelani S, Giannopoulos S, Machairas N, Kykalos S, Koliakos N (2021). Management of Morgagni's Hernia in the Adult Population: A Systematic Review of the Literature. World J Surg.

